# A suite of modular, all-synthetic suicide vectors for allelic exchange mutagenesis in multidrug resistant *Acinetobacter* strains

**DOI:** 10.1186/s12866-023-02844-7

**Published:** 2023-05-18

**Authors:** Alaska Pokhrel, Liping Li, Francesca L. Short, Ian T. Paulsen

**Affiliations:** 1 School of Natural Sciences, Macquarie University, Sydney, NSW 2109 Australia; 2 ARC Centre of Excellence in Synthetic Biology, Macquarie University, Sydney, NSW 2109 Australia; 3 Department of Microbiology, Monash University, 19 Innovation Walk, Clayton, VIC 3800 Australia

**Keywords:** *Acinetobacter baumannii*, MDR, Genetic manipulation, Allelic-exchange mutagenesis, Homologous recombination, SEVA, Conjugation, Scar-less deletion

## Abstract

**Background:**

*Acinetobacter baumannii* is an opportunistic human pathogen that causes a variety of infections in immunosuppressed individuals and patients in intensive care units. The success of this pathogen in nosocomial settings can be directly attributed to its persistent nature and its ability to rapidly acquire multidrug resistance. It is now considered to be one of the top priority pathogens for development of novel therapeutic approaches. Several high-throughput techniques have been utilised to identify the genetic determinants contributing to the success of *A. baumannii* as a global pathogen. However, targeted gene-function studies remain challenging due to the lack of appropriate genetic tools.

**Results:**

Here, we have constructed a series of all-synthetic allelic exchange vectors – pALFI1, pALFI2 and pALFI3 – with suitable selection markers for targeted genetic studies in highly drug resistant *A. baumannii* isolates. The vectors follow the Standard European Vector Architecture (SEVA) framework for easy replacement of components. This method allows for rapid plasmid construction with the mutant allele, efficient conjugational transfer using a diaminopimelic acid-dependent *Escherichia coli* donor strain, efficient positive selection using the suitable selection markers and finally, sucrose-dependent counter-selection to obtain double-crossovers.

**Conclusions:**

We have used this method to create scar-less deletion mutants in three different strains of *A. baumannii*, which resulted in up to 75% deletion frequency of the targeted gene. We believe this method can be effectively used to perform genetic manipulation studies in multidrug resistant Gram-negative bacterial strains.

**Supplementary Information:**

The online version contains supplementary material available at 10.1186/s12866-023-02844-7.

## Background

*Acinetobacter baumannii* is one of the most prevalent multidrug-resistant hospital pathogens globally. *A. baumannii* is now considered a global threat in clinical settings due to its propensity to rapidly acquire antimicrobial resistance determinants, and its ability to persist on various abiotic surfaces facilitating nosocomial transmission [1–3]. Due to widespread multidrug resistance (MDR) in this species, the WHO and CDC have listed carbapenem-resistant *A. baumannii* as a top priority pathogen for research and development of new antimicrobial treatments [4, 5].

In order to combat this problematic pathogen, it is important to identify the genetic determinants contributing to its success. However, studies of gene knockouts have been limited due to the paucity of advanced genetic manipulation tools. While there are many well-established gene modification techniques that work broadly in Gram-negative bacteria, gene manipulation in *A. baumannii* is still challenging. This is especially true for extensively drug resistant clinical isolates, which are resistant to most of the antibiotics that are commonly used in laboratory for selection [6–9, 13, 14]. Given the rise of highly drug resistant isolates of *A. baumannii*, studies involving MDR and virulent isolates will best reflect the molecular dynamics behind *A. baumannii* pathogenesis. Therefore, it is of utmost importance to develop genetic manipulation tools that are suitable for use in highly drug resistant strains of *A. baumannii.*

Allelic exchange mutagenesis is an efficient method used in bacterial genome editing. This method enables construction of gene knockouts, knock-ins and other site-directed mutations in a wide range of bacterial species [10, 11]. To facilitate allelic exchange, a suicide vector is constructed with a copy of the mutant allele flanked by regions adjacent to the gene of interest that are identical to the recipient chromosome. The suicide vectors for allelic exchange usually contain an origin of transfer (OriT) and therefore conjugation is most often used to introduce the plasmid carrying the mutant allele to the recipient strain. Allelic exchange mutagenesis is a two-step procedure, where a site-specific chromosomal integration event mediated by homologous recombination occurs in the first step, followed by the plasmid backbone excision from the chromosome via a second crossover event, which results in allelic exchange. The first homologous recombination event, also known as a single crossover, results in the recipient acquiring resistance to the antibiotic marker encoded on the suicide vector. The antibiotic-resistant merodiploids are selected and then subject to a counter-selection method to isolate the double-crossovers [10, 11]. The majority of suicide vectors used in Gram-negative bacteria include a *sacB* gene from *Bacillus subtilis* encoding levansucrase for counter-selection. Levansucrase is an enzyme that confers sucrose sensitivity in Gram-negative bacteria by catalysing the hydrolysis of sucrose into glucose and the toxic product levan in the periplasm [6, 11, 12]. Double crossover mutants are isolated by growing the single crossover mutants in the presence of sucrose, as only those bacteria that have lost the *sacB* gene can grow under this condition. Although allelic exchange mutagenesis is one of the most common techniques to create marker-less mutants in Gram-negative bacteria [10, 11], and has been previously used in *A. baumannii* [13, 14], many widely used allelic exchange vectors lack suitable selection markers for MDR strains and are also difficult to modify or edit due to their lack of suitable restriction sites and complex architectures. Therefore, this method is still not easily applied to many clinical *A. baumannii* isolates.

**Fig. 1:**
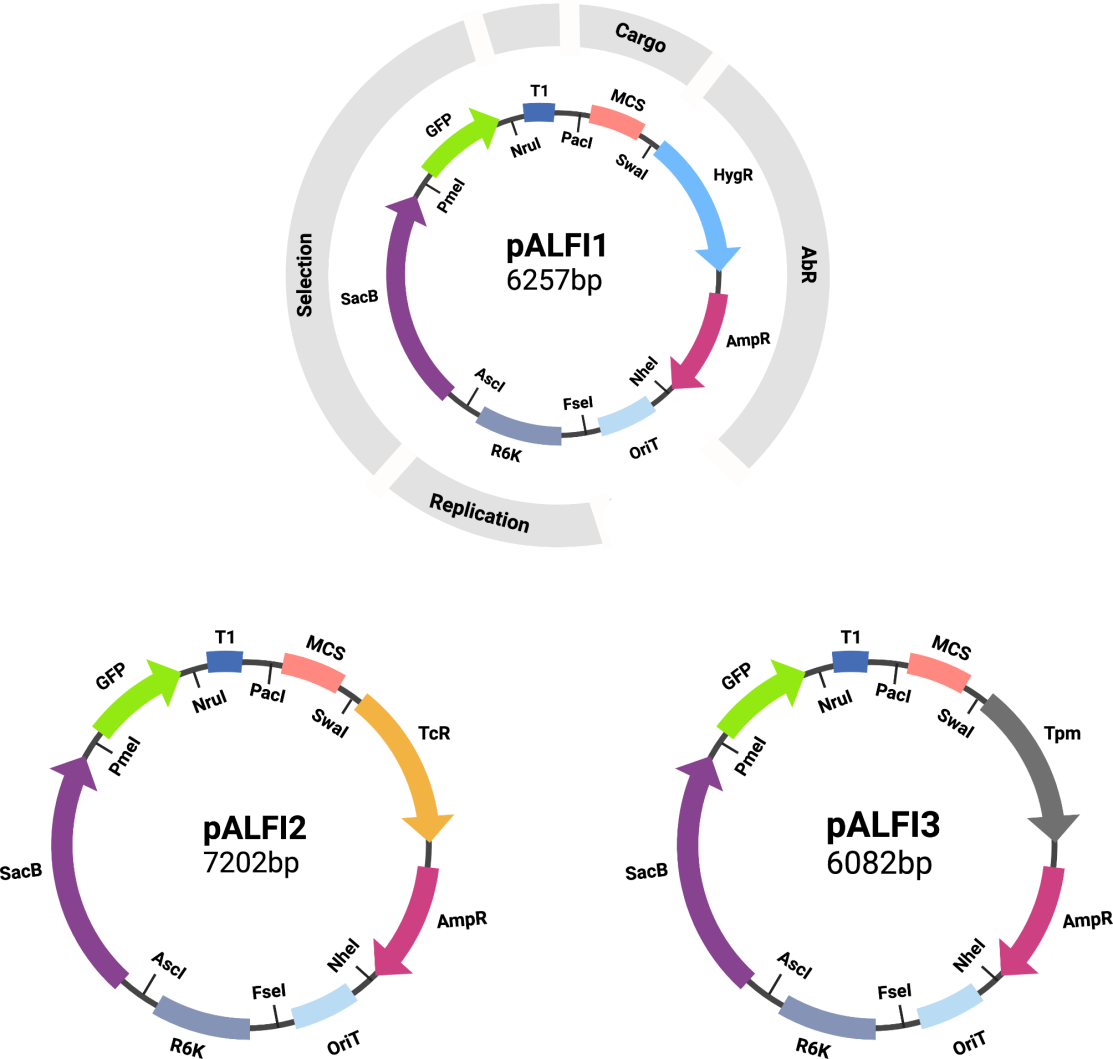
Map of pALFI vectors showing how they follow the SEVA architecture. The cargo segment includes the MCS (multi cloning site); Ab^R^ segment includes HygR (hygromycin resistance cassette) and AmpR (ampicillin resistance cassette); the replication segment includes R6K (pir-dependent origin of replication); an addition selection segment (which is not standard in SEVA vectors) includes SacB (levansucrase gene) and GFP (green fluorescent protein); The pALFI vectors also include an OriT (origin of conjugational transfer) and T1 (terminator). Each genetic element is flanked with unique restriction sites. The HygR resistance marker in pALFI1 is replaced with TcR in pALFI2 and with Tpm in pALFI3 (Created with BioRender.com).

In this study, we have built and validated a set of all-synthetic, Standard European Vector Architecture (SEVA)-based suicide vectors with varied selection markers, with the specific aim of enabling allelic exchange mutagenesis of MDR *A. baumannii* strains (Fig. 1). Use of these vectors provided an efficient and quick method for genetic manipulations in various drug susceptible and MDR *A. baumannii* strains with deletion frequencies up to 75%.

## Results and discussion

We first designed a series of vectors – pALFI1, pALFI2 and pALFI3 – with broadly useful selection markers for MDR *A. baumannii* strains, as well as properties allowing easy modification in future studies. The pALFI vectors follow the Standard European Vector Architecture (SEVA)[15] for easy replacement of components, and contain a choice of hygromycin, tetracycline or tellurite resistance markers (Fig. 1). The three vectors contain all genetic elements commonly present in suicide vectors: the R6K replication origin for pir-dependent replication, *oriT* for transfer by conjugation, the *sacB* gene for selection of double crossovers, and the standard SEVA multiple cloning site for introduction of allelic exchange cassettes [Ortiz-Martín et al., 2006; Biswas, 2015; Hmelo et al., 2015; Cianfanelli et al., 2020].

We created three different vector derivatives to expand the usability of these vectors in susceptible as well as MDR *A. baumannii* strains. The pALFI1 vector contains the *hygR* gene encoding an aminoglycoside phosphotransferase that confers resistance to hygromycin, which can be used in strains that are resistant to other aminoglycosides. The *tetA* gene conferring tetracycline resistance is included in pALFI2 for genetic manipulation in susceptible *A. baumannii* strains, such as ATCC17978. A non-antibiotic selection marker, Tpm (thiopurine-S-methyltranferase), conferring resistance to tellurite, is included in pALFI3 for use when there are no suitable antibiotic resistance markers. The pALFI- vectors also encode an ampicillin resistance marker, which allows for the selection of transformants in *E. coli*. We used ampicillin selection for plasmid cloning and propagation, and the other markers only for positive selection of trans-conjugants, thus limiting the use of toxic or expensive selection agents. Vector construction and mutagenesis of target strains was performed using *E. coli* strain Jke201 – a pir-carrying, diaminopimelic acid (DAP)-auxotrophic donor strain that allows for replication of plasmids containing an R6K origin and the subsequent removal of donor strain on any growth medium not supplemented with DAP [12, 16].

In order to test the efficiency of the pALFI vectors, we deleted the *aceI* efflux pump gene in two different *A. baumannii* strains: AB5075_UW [17] using both pALFI1 and pALFI3, and AB0057[18](Table 1) using pALFI1. AceI is a member of the proteobacterial antimicrobial compound efflux (PACE) family of transport proteins and is highly conserved across *A. baumannii* strains [19, 20]. Similarly, we used pALFI3 to create a deletion mutant lacking sensor histidine kinase gene, BAL062_00580, in another highly MDR strain BAL062 [21, 22]. Initial susceptibility testing showed that the optimal concentrations for selection were 250 µg/ml hygromycin in AB5075_UW and AB0057, and 50 µg/ml potassium tellurite in BAL062 and AB5075_UW.

**Table 1:** *A. baumannii* strains used in the study

Strains	Source	Antibiotic resistance	Vector (selection agent used for knockout)	Deletion frequency
AB5075_UW	MDR *A. baumannii* wound isolate recovered from a patient at the Walter Reed Army Medical Center, USA [17, 26, 27].	Resistant to beta-lactams, aminoglycosides (sensitive to hygromycin), fluoroquinolones, chloramphenicol, trimethoprim, sulfonamides	pALFI1 (Hygromycin 250 µg/ml) and pALFI3 (Tellurite 50 µg/ml)	75% (pALFI1) and 40% (pALFI3)
AB0057	MDR *A. baumannii* bloodstream isolate recovered from a patient at the Walter Reed Army Medical Center, USA [18, 28].	Resistant to beta-lactams, aminoglycosides (sensitive to hygromycin), fluoroquinolones, tetracycline, chloramphenicol, trimethoprim, sulfonamides	pALFI1 (Hygromycin 250 µg/ml)	50%
BAL062	MDR *A. baumannii* Bronchoalveolar lavage isolate recovered from patient with ventilator-associated pneumonia at the Hospital for Tropical Diseases in Ho Chi Minh City, Vietnam [21, 22].	Resistant to beta-lactams, aminoglycosides, fluoroquinolones, cephalosporins, trimoxazole, imipenem	pALFI3 (Tellurite 50 µg/ml)	50%

The counter-selection plates containing 5% sucrose were found to contain some sucrose-resistant merodiploids, which may have occurred due to inactivating mutations in the *sacB* gene [11]. An additional selection step, where colonies from sucrose plates were transferred into fresh sucrose as well as selective agar plates (containing hygromycin or tellurite) helped in the identification of colonies in which plasmid excision had not occurred. A total of 20 colonies that grew on sucrose plates but not on selection plates were screened by colony PCR. Deletion frequencies of 75% and 50% was observed with pALFI1 in AB5075_UW and AB0057, respectively. The use of pALFI3 in AB5075_UW and BAL062 resulted in deletion frequencies of 40% and 50% respectively (Supplementary Fig. 2). The deletion frequencies were calculated by dividing the number of positive colonies per strain by the total number of colonies screened.

**Fig. 2:**
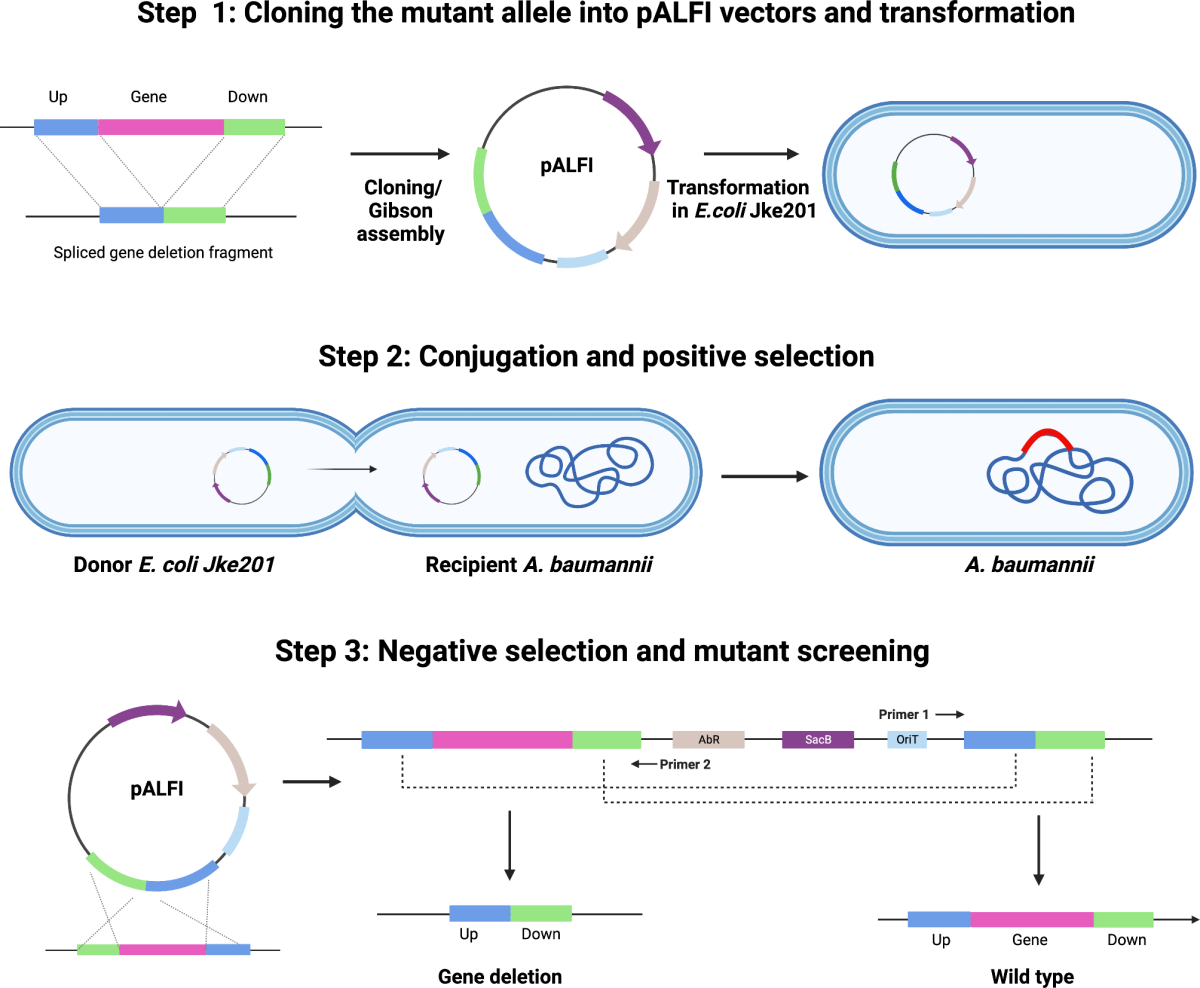
**An overview of allelic exchange mutagenesis protocol using pALFI vectors** Mutant allele is first cloned into an all elic exchange vector (pALFI) and transformed into *E. coli* Jke201 or another suitable donor strain for biparental mating. This step can ideally be completed in two working days. In the second step, the knockout vector is introduced into the *Acinetobacter* recipient strain by conjugation and screened for single-crossover mutants (positive selection). This step can be completed in 2–3 days. In the final step, single crossovers are transferred onto sucrose plates (negative selection) for mutant screening, which can be completed in 4 working days. Typically, the whole process can be completed in 8–10 working days. A detailed step by step protocol is included as the supplementary material S1 (Created with BioRender.com).

In theory, the frequency of allelic exchange using suicide vectors would be expected to be around 50%, however this is often not the case. The deletion frequencies using the pALFI vectors was observed to be between 40 and 75%, demonstrating the reasonable efficiency of this method. Taken together, the whole method from cloning the mutant allele in suicide vector to obtaining a knockout mutant took a total of 8–10 days (Fig. 2).

## Conclusions

Genetic manipulation enables understanding of gene-phenotype relationships and allows for the identification of novel therapeutic targets to combat problematic pathogens, such as *A. baumannii*. The allelic exchange vectors generated in this study were found to be efficient for obtaining scar-less mutants in MDR *A. baumannii* isolates. We believe that this method and the allelic exchange vectors can also be effectively used for gene-function studies in other highly multidrug resistant bacterial species, in which genetic manipulation is troublesome.

## Methods

### Bacterial strains, growth media and conditions

Bacterial strains were cultured in Luria-Bertani (LB) media at 37 °C unless otherwise stated. *E. coli* Jke201 was used for plasmid propagation and as a donor strain for conjugation [12], and was cultured in the presence of 100µM of diaminopimelic acid (DAP) (Sigma Aldrich). *A. baumannii* strains AB5075_UW, AB0057, BAL062 were included in this study as recipient strains (Tables 1 and 2).

**Table 2:** *E. coli* strain and plasmids used in the study

Strain/Plasmids	Description	Supplementation/ Selection	Source
Jke201	Pir-carrying, diaminopimelic acid (DAP)-auxotrophic *E. coli* donor strain	diaminopimelic acid (DAP)	[12]
pFOKT	Suicide vector	Kanamycin and tellurite	[12]
pKNG101_Tc	Suicide vector	Tetracycline	[24]
pALFI1	Suicide vector	Hygromycin and ampicillin	This study
pALFI2	Suicide vector	Tetracycline and ampicillin	This study
pALFI3	Suicide vector	Tellurite and ampicillin	This study

Chemically competent cells for *E. coli* Jke201 were prepared using the calcium chloride/MES (2-(N-morpholino) ethanesulfonic acid) method. Briefly, cells were grown to mid exponential phase (OD_600nm_ 0.4–0.6) from overnight cultures in the presence of 15mM MgCl_2_. Bacterial cultures were chilled on ice for 40 min before harvesting. The cell pellets were washed twice in 10ml ice-cold solution A (50 mm CaCl_2_, 10mM MnCl_2_, 10mM MES at pH 6.3). The cells were then resuspended in 1.5ml solution A containing 15%(v/v) glycerol and 100 µl aliquots were either used directly or frozen and stored at -80 °C.

Transformation was performed using the heat shock method [23]. *E. coli* transformants were selected in the presence of 100 µg/ml ampicillin (Sigma Aldrich). For positive selection of trans-conjugants, a final concentration of 250 µg/ml hygromycin (Invitrogen) or 10 µg/ml tetracycline (Amresco) or 50 µg/ml potassium tellurite (Sigma Aldrich) was used. For counter-selection, fresh low-salt LB plates (10 g/L tryptone, 5 g/L NaCl, 5 g/L yeast extract, 15 g/L agar) with 5% sucrose (Sigma Aldrich) were prepared.

### Construction of suicide vectors

Suicide vector pALFI was constructed by combining the origin of conjugational transfer (OriT), origin of replication (R6K), and multiple cloning site (MCS) from the SEVA database [15]. The *sacB* gene sequence with its promoter region was obtained from pKNG101_Tc [24]. pALFI1 was generated by combining the hygromycin (HygR) and ampicillin (AmpR) resistance cassettes and a reporter gene (green fluorescent protein, GFP). The GFP gene, preceded by the *amvR* promoter [25] was intended for use as a second selection marker for single crossovers, however the signal was not high enough under normal growth conditions to reliably indicate colonies containing the plasmid. Each segment of the pALFI suicide vector is flanked with unique restriction sites, adopting the SEVA standard [15] (Fig. 1). The plasmid sequence of pALFI1 was submitted for plasmid synthesis (GENEWIZ). Once the pALFI1 plasmid was synthesized, pALFI2 was generated by replacing *hygR* with *tetA* with its native regulator *tetR* from *A. baumannii* AB0057. pALFI3 was generated by replacing *hygR* with *tpm* (thiopurine-S-methyltranferase) conferring resistance to tellurite from pFOKT [12](Supplementary Fig. 1). The TcR and Tpm markers were cloned using the *Swa*I/*Sca*I restriction sites. Plasmids used in this work are listed in Table 2. Primers used to amplify the tetracycline and tellurite resistance cassettes are listed in Table S1.

### Cloning the mutant allele into the suicide vector

In order to amplify the knockout fragment containing approximately 700 bp upstream and downstream region of targeted gene, flanking primers with 15-18 bp overlap (Table S1) were designed. The upstream and downstream regions were first amplified using the high-fidelity Platinum SuperFi PCR master mix (Invitrogen) and purified from a 1% agarose gel (QiAquick gel extraction kit, Qiagen). The fragments were then spliced together by overlap extension PCR and gel purified. Plasmids were isolated from overnight cultures using Wizard Plus SV miniprep kit (Promega). Plasmid and the knockout fragment were digested using *Spe*I-HF or *Bam*HI-HF (New England Biolabs) for 2 h at 37 °C and gel purified. The restriction digested knockout fragment and plasmid were ligated together using T4 DNA ligase (New England Biolabs) and transformed into *E. coli* Jke201 using the heat-shock method. After a heat shock, the cells were resuspended in pre-warmed LB broth supplemented with 100µM DAP and incubated at 37 °C for 2 h before plating on an ampicillin (100 µg/ml) selection plate. Transformants were screened by colony PCR using the primers flanking the upstream and downstream regions of the targeted gene (Table S1).

### Conjugation, positive selection and counterselection

Recipient *A. baumannii* strains AB5075_UW, AB0057, and BAL062 were streaked on fresh LB agar plates containing no antibiotics and incubated overnight at 37°C. The donor strain, *E. coli* JKe201 was streaked on a fresh LB agar plate supplemented with 100µM DAP and 100 µg/ml ampicillin and incubated overnight at 37°C. Cells from each plate were resuspended in sterile phosphate buffered saline (PBS) and adjusted to an optical density (OD_600nm_) of 40 for the donor strain and 20 for the recipient strains. Equal volumes of recipient and donor strains were mixed and spotted on a very dry LB agar plate supplemented with 100µM DAP. The conjugation patch was left to dry and incubated at 37°C for 2 h.

For positive selection, a loopful of cells from the conjugation patch was streaked on LB agar plate containing 250 µg/ml hygromycin (pALFI1) or 20–100 µg/ml potassium tellurite (pALFI3) and incubated at 37°C overnight. The conjugation plate was incubated at 28°C overnight and a loopful of cells was again transferred onto the selective LB agar plates the next day (16-18 h). The selection conditions were optimised for each *A. baumannii* strains to avoid background growth. The single crossover mutants were screened by colony PCR to confirm the presence of the resistance cassette.

For counter-selection, at least three trans-conjugant colonies were selected and streaked on freshly prepared low-salt LB agar plates containing 5% sucrose. The plates were incubated at room temperature (RT) for 24-48 h until well defined colonies were obtained. The colonies from sucrose plates were patched onto a fresh selective LB agar plate as well as a fresh LB agar plate supplemented with sucrose to confirm the excision of the plasmid backbone. The selection plates were incubated at 37°C and the sucrose plates were incubated at RT. Colonies that grew on the LB sucrose plate but not on the selective LB plate were screened by PCR to identify the knockout mutants. Genomic DNA was extracted from the deletion mutants using the DNeasy ultraclean microbial kit (Qiagen) and mutants were confirmed by genome sequencing. A detailed step by step protocol is included as the supplementary material S1.

## Electronic supplementary material

Below is the link to the electronic supplementary material.

**Supplementary material S1**: Step by Step protocol. **Supplementary Figure 1**: ***tetA and tpm gene schematics. A***. tetA with its native regulator tetR conferring resistance to tetracycline, amplified from *A. baumannii* AB0057 as incorporated in pALFI2. B. *tpm* gene with its promoter conferring resistance to tellurite, amplified from pFOKT plasmid as incorporated in pALFI3. **Supplementary Figure 2**: **Verification of gene deletions by colony PCR**. Twenty colonies were screened per strain and per vector combination used. Colonies were considered positive for *aceI* gene deletion if a band of approximately 200bp was detected. The length of full *aceI* gene amplicon (positive control) is approximately 450 bp. Control samples (both positive and negative) were also included for reference. Deletion frequencies of 75% and 50% was observed with pALFI1 in AB5075_UW and AB0057, respectively for *aceI* gene deletion. The use of pALFI3 in AB5075_UW for *aceI* deletion resulted in 40% deletion frequency. **Table S1**: Primers used in the study.

## Data Availability

All data generated or analysed during this study are included in this published article [and its supplementary information files].
