# Supplementary material for: A suite of modular, all-synthetic suicide vectors for allelic exchange mutagenesis in multidrug resistant Acinetobacter strains
Source: BMC Microbiol. 2023 May 18;23:137. doi: 10.1186/s12866-023-02844-7 (PMC10193806; doi:10.1186/s12866-023-02844-7)
Supplement: Supplementary file 1 — Supplementary material S1: Step by Step protocol. Supplementary Figure 1: tetA and tpm gene schematics. A. tetA with its native regulator tetR conferring resistance to tetracycline, amplified from A. baumannii AB0057 as incorporated in pALFI2. B. tpm gene with its promoter conferring resistance to tellurite, amplified from pFOKT plasmid as incorporated in pALFI3. Supplementary Figure 2: Verification of gene deletions by colony PCR. Twenty colonies were screened per strain and per vector combination used. Colonies were considered positive for aceI gene deletion if a band of approximately 200bp was detected. The length of full aceI gene amplicon (positive control) is approximately 450 bp. Control samples (both positive and negative) were also included for reference. Deletion frequencies of 75% and 50% was observed with pALFI1 in AB5075_UW and AB0057, respectively for aceI gene deletion. The use of pALFI3 in AB5075_UW for aceI deletion resulted in 40% deletion frequency. Table S1: Primers used in the study. [file 12866_2023_2844_MOESM1_ESM.docx]

**Title:**

**A suite of modular, all-synthetic suicide vectors for allelic exchange mutagenesis in multidrug resistant *Acinetobacter* strains**

Alaska Pokhrel^1^, Liping Li^1,2^, Francesca L. Short^1,3^, Ian T. Paulsen^1,2^

**Supplementary material S1:** Step by Step protocol

*Step 1: Cloning the mutant allele into an allelic exchange vector (2 days)*

- 1. Design the knockout fragment. The knockout fragment should contain between 600 -1000 bases upstream and downstream flanking the gene of interest.
  2. Either have the knockout fragment synthesised or amplify and splice together the upstream and downstream fragments by overlap PCR.
  3. Clone the knockout fragment into the desired pALFI vector.
  4. Transform the cloning mixture into *E. coli* Jke201 or another suitable donor strain for biparental mating. The Jke201 donor strain requires DAP supplementation (100 µM).
  5. Use colony PCR to screen for correct clones containing the knockout fragment.

*Step 2: Conjugation to introduce the knockout vector into the Acinetobacter recipient strain and positive selection (2-3 days)*

- 1. Streak out the donor strain and recipient strain on LB agar + antibiotics/supplements and grow overnight at 37˚C.
  2. Resuspend cells from each plate in sterile PBS to an OD_600nm_ of 40 (donor) or 20 (recipient).
  3. Spot 25 µl donor, 25 µl recipient and 50 µl combined donor and recipient onto the surface of a very dry LB + DAP (100 µg/ml) plate for the conjugation. Allow patches to dry completely.
  4. Incubate plate face up at 37˚C for 2 hours (Time point 1), transfer to room temperature and incubate overnight (Time point 2).
  5. At time points 1 and 2, streak out a loopful of cells from the conjugation patch and each control patch onto selective agar plates and incubate overnight at 37˚C.
  6. Re-streak 4 putative single-crossover mutants on selective media and grow overnight. Optional: confirm presence of the resistance cassette by PCR, stock single-crossover mutants at -80˚C.

*Step 3: Counterselection and mutant screening (4 days)*

- 1. Streak out the single crossovers onto low-salt LB plates supplemented with 5% sucrose. Incubate at room temperature for 2-3 days until you see well defined colonies.
  2. Patch colonies from the sucrose plates onto fresh LB + selection and LB + Sucrose plates to confirm loss of the vector. Grow sucrose plates at RT and selection plates at 37˚C.
  3. Test clones that grow on sucrose but not selection plates by PCR to confirm loss of the gene of interest.

**Supplementary Figure 1:**


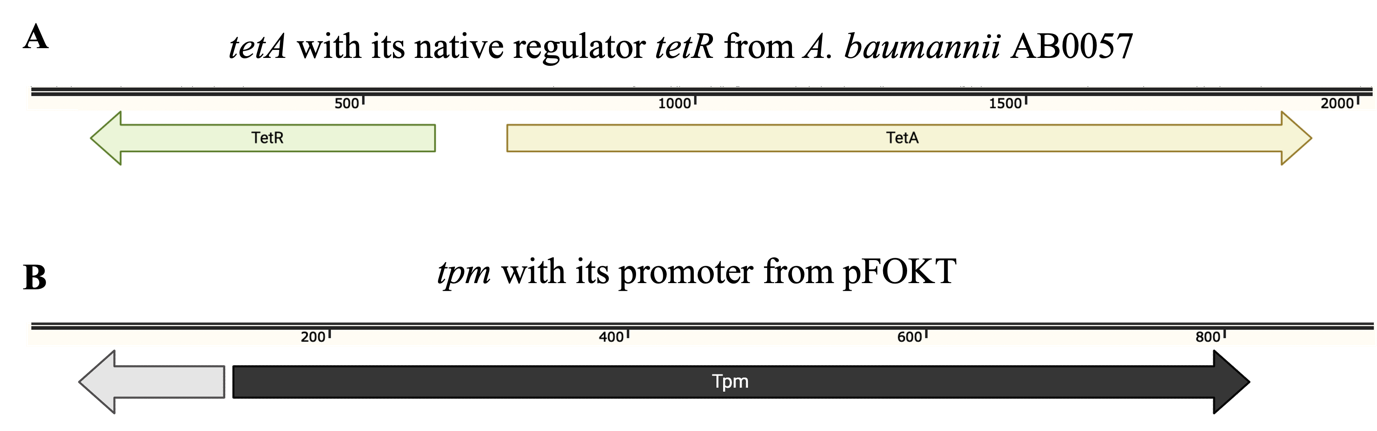


***tetA* and *tpm* gene schematics.** **A.** *tetA* with its native regulator *tetR* conferring resistance to tetracycline, amplified from *A. baumannii* AB0057 as incorporated in pALFI2. **B.** *tpm* gene with its promoter conferring resistance to tellurite, amplified from pFOKT plasmid as incorporated in pALFI3.

**Supplementary Figure 2:**

**Verification of gene deletions by colony PCR.** Twenty colonies were screened per strain and per vector combination used. Colonies were considered positive for *aceI* gene deletion if a band of approximately 200bp was detected. The length of full *aceI* gene amplicon (positive control) is approximately 450 bp. Control samples (both positive and negative) were also included for reference. Deletion frequencies of 75% and 50% was observed with pALFI1 in AB5075_UW and AB0057, respectively for *aceI* gene deletion. The use of pALFI3 in AB5075_UW for *aceI* deletion resulted in 40% deletion frequency.

**Table S1:** Primers used in the study.

| **Primer name** | **Sequence** | **Description** |
| --- | --- | --- |
| aceI_primerA | ACTAGTATAACCGGAATTAAGGCTTCACGT | Forward primer to amplify upstream region of *aceI* |
| aceI_primerB | TCTGGGTGCTTATTTACTTGGATAATGTGCATT | Reverse primer to amplify upstream region of *aceI* |
| aceI_primerC | GCACATTATCCAAGTAAATAAGCACCCAGATGG | Forward primer to amplify downstream region of *aceI* |
| aceI_primerD | ACTAGTACTGCAACAATGACAGCGGGT | Reverse primer to amplify downstream region of *aceI* |
| aceIKO_PCRf | AGCAATCGACATACTCACAGCAGA | Forward *aceI* external primer to screen for knockouts |
| aceIKO_PCRr | ATTTGCTGCAACTGGTGGTCGTAT | Reverse *aceI* external primer to screen for knockouts |
| BAL062_00580_primerA | GAGTGGATCCCTTTGGTTCCGACCCTCGG | Forward primer to amplify upstream region of BAL062_00580 and to screen for knockouts |
| BAL062_00580__primerB | TAGTAGGAAAAGGGAGCCCGCCTCACAGATTTT | Reverse primer to amplify upstream region of BAL062_00580 |
| BAL062_00580__primerC | ATCTGTGAGGCGGGCTCCCTTTTCCTACTACTACTTGAA | Forward primer to amplify downstream region of BAL062_00580 |
| BAL062_00580__primerD | GAGTGGATCCACCGTTGGTTTCGCCTTGC | Reverse primer to amplify downstream region of BAL062_00580 and to screen for knockouts |
| pALFI_M13F | GGTTTTCCCAGTCACGAC | Forward M13 primer flanking MCS in pALFI vectors |
| pALFI_M13R | AGCGGATAACAATTTCACAC | Reverse M13 primer flanking MCS in pALFI vectors |
| pALFI1_HygR_F | GAAAAAGCCTGAACTCACCGC | Forward primer to amplify *hygR* resistance gene in pALFI1 |
| pALFI1_HygR_R | CGCTCATTAGGCGGGCTACTA | Reverse primer to amplify *hygR* resistance gene in pALFI1 |
| TetR_ SwaIF | ATTTAAATTGAGTGGGTTGCGCTCCGG | Forward primer to amplify *tetR* and *tetA* regions in *A. baumannii* AB0057 |
| TetR_ScaIR | AGTACTCCCGCATGGCATAGGCCT | Reverse primer to amplify *tetR* and *tetA* regions in *A. baumannii* AB0057 |
| Tpm_SwaIF | ATTTAAATGCGGGACTCTGGGGTTCGA | Forward primer to amplify *tpm* with its promoter region in pFOKT |
| Tpm_ScaIR | AGTACTCCTACCGCGAAAGGTTTTGC | Reverse primer to amplify *tpm* with its promoter region in pFOKT |
